# Effects of Motor Mental Imagery Training on Tennis Service Performance during the Ramadan Fasting: A Randomized, Controlled Trial

**DOI:** 10.3390/nu12041035

**Published:** 2020-04-09

**Authors:** Sofien Fekih, Mohamed Sami Zguira, Abdessalem Koubaa, Liwa Masmoudi, Nicola Luigi Bragazzi, Mohamed Jarraya

**Affiliations:** 1 Higher Institute of Sport and Physical Education of Gafsa, Gafsa 2100, Tunisia; fkih2007@hotmail.com (S.F.); sami-zguira@hotmail.fr (M.S.Z.); abdessalemkoubaa@gmail.com (A.K.); 2 Institute of Sport and Physical Education of Ksar Said, University of Manouba, Manouba 2010, Tunisia; 3 Research Unit: Education, Motricity, Sports and Health, (EM2S, UR15JS01), Higher Institute of Sport and Physical Education of Sfax, University of Sfax, Sfax 3100, Tunisia; liwa.masmoudi@yahoo.fr (L.M.); jarrayam@yahoo.fr (M.J.); 4 Department of Physiology and Lung Function Testing, Faculty of Medicine Ibn-El-Jazzar, University of Sousse, Sousse 4000, Tunisia; 5 Laboratory of Pharmacology, Faculty of Medicine of Sfax, University of Sfax, Sfax 3100, Tunisia; 6 Department of Neuroscience, Rehabilitation, Ophthalmology, Genetics, Maternal and Child Health (DINOGMI), Section of Psychiatry, Genoa University, 16132 Genoa, Italy; 7 Postgraduate School of Public Health, Department of Health Sciences (DISSAL), Genoa University, 16132 Genoa, Italy; 8 Laboratory for Industrial and Applied Mathematics (LIAM), Department of Mathematics and Statistics, York University, Toronto, ON M3J1P3, Canada; 9 Research Laboratory Sport Performance Optimization, National Centre of Medicine and Sciences in Sport (CNMSS), Tunis 2000, Tunisia

**Keywords:** intermittent fasting, Ramadan fasting, tennis, service performance, motor mental imagery training, sport psychology

## Abstract

The objective of the present study is to analyze the effects of motor mental imagery training on tennis service performance among tennis athletes who fast during Ramadan. Participants were 38 young male tennis players, randomly divided into two groups: Imaging Training (IMG, *n* = 18) and control group (CG, *n* = 20). The CG has watched videos on the history of the Olympic Games, while IMG has followed a training program in motor imagery. The performance of the tennis service was obtained by the product between accuracy and speed of typing (accuracy × average speed of all shots (km/h)). The effect of group/time interaction (*p* < 0.01) was identified for all performance indicators (accuracy, running speed and performance (speed × precision)), with improvement only in IMG (*p* = 0.01). The results showed that motor imagery training could be an effective strategy for mitigating/counteracting the negative effects of Ramadan on the tennis service performance.

## 1. Introduction

The fasting of Ramadan is an important religious tradition observed in the Muslim world, in which Muslims in good health are required to abstain from drinking, eating, and having sexual intercourse, from early dawn to sunset. The duration and the effects of the Ramadan fasting depend on a series of parameters, including the climatic conditions and the geographical location. Based on these variables, Ramadan fasting can last up to 18 hours per day in summer in hot regions.

From the existing scholarly literature, it is well-known that Ramadan-induced sleep-wake disruptions and changes in dietary habits can have a profound effect on athletic performance [1]. In recent years, the fasting of Ramadan has occurred during the summer, with many national tennis events in Tunisia (for example, regional tournaments, championship games and tennis shots) and international ones (for example, the tennis tournament Roland Garros in 2017), taking place during this month. Due to this coincidence between hot temperature and fasting, Muslim tennis players fasting during the month of Ramadan should pay particular attention to the high dehydration caused by fasting during the day, the hot weather conditions and their interaction, which can affect their sports performance [2]. Several studies have shown that the effects of Ramadan fasting on athletic performance depend on an array of various factors, such as the physical conditions of the participants, whether or not they are able to maintain the same training load compared to pre-Ramadan, and the nature of the effort provided [3,4,5,6,7].

According to the main characteristics of the game, tennis players perform intermittent muscular actions, alternating intense and active recovery moments during the match [8,9,10]. To perform well in tennis, aerobic fitness, sprint rehearsal ability, agility and muscle power are among the necessary skills [11,12]. Tennis matches can last, on average, up to two hours or even more [10]. Furthermore, a study [13] has reported that athletes fasting while training or participating in competitions longer than 30 minutes, such as tennis players, may face particular difficulties when the ambient temperature is high. To succeed in tennis, players need good technical skills (forehand, backhand, lob, smash and service) and physical skills. Thus, service is one of the essential keys to have a good performance during a match, since it will result in a direct score [14]. In the effort to achieve a greater efficiency, tennis service should combine two fundamental components like speed and accuracy to increase the likelihood of winning and the number of points gained [11,14]. In addition, a study has shown that approximately 25% of tennis points come from service [15]. 

Conditions impacting on speed and accuracy can be, as such, detrimental in terms of performance, suggesting that the fasting of Ramadan could have negative effects on tennis performance and on related aspects. Adequate physical and technical training can improve the performance of tennis service under normal conditions [14] and may help to counteract or, at least, mitigate the negative effects of Ramadan on the performance of tennis players at the level of service.

Therefore, in order to properly deal with the effects of the fasting of Ramadan, sports coaches and managers should find effective strategies aimed at improving athletic performance among fasting Muslim athletes. Various studies have shown that the incorporation of a motor imaging training module into the athlete training program on a regular basis could help optimize athletic performance, for example on free throws [16], tennis service [17,18] and sports disciplines and physical activities requiring strength [19]. In fact, training incorporating motor imagery modules is an alternative widely used by sports psychologists and coaches to improve the physical performance of athletes [17,20,21,22]. According to a study [23], motor imagery can be realized from an internal perspective (use of “I”), with the athlete being directly involved in the action and imagining himself with his body performing the gesture, feeling all the sensations provided (visual and kinesthetic). This is termed as “associated mental imagery”. Motor imagery can be realized also from an external perspective (use of “he”), with the sportsman being spectator of his action, and visualizing it. This is termed as “dissociated mental imagery”. 

To the best of our knowledge, there is a dearth of data concerning the effects of Ramadan fasting on performance achieved during high-intensity intermittent exercises/activities requiring significant physical and cognitive abilities, such as tennis matches performed in hot environments [13]. There is also a lack of studies assessing the effects of motor imagery training on the performance of tennis service during the month of Ramadan.

Therefore, this study was undertaken to fill this gap in the existing knowledge by carrying out a randomized, controlled trial with tennis athletes fasting during the month of Ramadan. This could help tennis coaches better understand the chronic effects of Ramadan fasting on service performance and, as such, choose whether or not to incorporate a motor imagery training module in the routine training of Muslim athletes fasting during Ramadan. 

Two hypotheses have been formulated. First, we hypothesized that fasting during the month of Ramadan has a negative effect on the performance of tennis service (in terms of accuracy and speed). Second, we hypothesized that the motor imagery training program could counteract or mitigate the negative effects of the Ramadan fasting on the performance of tennis service (in terms of accuracy and speed).

## 2. Material and Methods

### Sample Size

The null hypothesis was formulated as H_0_: m_1_ = m_2_, whereas the alternative hypothesis was formulated as H_a_: m_1_ = m_2_ + *d*, where *d* is the difference between the two means and *n*_1_ and *n*_2_ are the sample sizes for the experimental and control groups, respectively, such as that *N* = *n*_1_ + *n*_2_. 

The total sample size was estimated using the following Formula (1) [24]:

(1) $$N=\frac{\left(r+1\right)\cdot {\left(Z_{\alpha /2}+Z_{1-\beta }\right)}^{2}\cdot \sigma^{2}}{r\cdot d^{2}}$$

where *Z_α_* is the normal deviate at the level of statistical significance = 1.64 (5% level of significance), *Z_1−β_* is the normal deviate at 1 − *β*% power with *β*% of type II error (0.84 at 80% statistical power); *r* is computed as *n*_1_/*n*_2_ that is to say the ratio of the sample size required for the two groups (*r* = 0.67 gives the sample size distribution as 1:1.5 for two groups). *σ* and *d* are the pooled standard deviation (SD). These two values were obtained from a previous study based on a similar hypothesis in a similar setting [18].

Thirty-eight young male tennis players volunteered to participate in this study, training regularly in tennis clubs for two hours per day, on average three times per week. To be included in the present research, given the criteria for inclusion of other surveys with racquet sports athletes [14,25], participants should: (a) be athletes practicing tennis for at least two years; (b) systematically training for at least six hours per week; and (c) be registered at the National Tennis Championship. Furthermore, they should be willing to fast throughout the entire month of Ramadan. These players were randomly allocated to two groups, an experimental group of mental imagery (IMG) of 18 players, and a control group (CG) of 20 players. There were no differences between the two groups in terms of sex distribution, mean age, height, body mass and number of years of experience. We also verified that none of the participants had experience in mental imagery training. Written, informed consent was obtained from all participants prior to data collection. Prior to the experiment, all participants had completed the French version of the revised motion imagery questionnaire [26]. The anthropometric data and the movement imaging questionnaire (MIQ-RS) scores for both groups (IMG and CG) are presented in Table 1.

The study protocol was reviewed in depth and fully approved by the ethical committee of the Farhat Hached Hospital, Sousse, Tunisia (protocol number ID IRB00008931-03). This study was conducted in accordance with the latest version of the Declaration of Helsinki. This is a randomized, controlled, four-week experimental study carried out during the month of Ramadan in 2017. Both groups (IMG and CG) completed the same Physical/Technical Training Plan, two hours per training session in the afternoon from 17.00 to 19.00. Registration code of the trial is PACTR202003492538049. 

During this study, athletes were asked to observe standardized calorie (Table 2) and hydric intake for the three days preceding each testing, to avoid dietary behaviors that could affect physical performance. Furthermore, we have instructed athletes to maintain the same sleeping hours as before the month of Ramadan. 

The CG watched videos about the history of the Olympic Games, while the IMG was provided with motor imagery training. Three weekly motor imaging training sessions were conducted per week, over a 48-h period, for a total of twelve sessions over a four-week period. The sessions were held at intervals of 30 min between the end of the motor imaging workout and the start of the physical/technical training session. All of the motor images training sessions lasted about ten minutes and were carried out in a quiet environment (near the tennis court), where the athletes wore the clothes they usually wore to compete in tennis matches [27]. The use of videos is necessary to improve the motor images, as this facilitates the observation in the context of the sport, stimulating specific brain areas. This study adopted a specific cognitive imagery training, in which athletes were asked to imagine themselves while performing tennis service, in agreement with a previous study [17]. The method adopted was the following: (a) the athlete was asked to imagine a first-person situation; (b) the athlete was asked to imagine the task performed at speeds close to reality, with actions interspersed by an interval of about ten seconds (c) the athlete was asked to imagine positive situations during a competition; and, finally, (d) the athlete was asked to reproduce emotions (including anxiety and mood) similar to those felt during competitions [27,28].

On ten fantasy screens of tennis service, participants were invited to produce information based on the chosen technique and the extent of perception and emotions of anxiety and mood, during the process of imagination. Then, the researcher, who was in charge of the realization of the protocol of the training in motor imagery, informed the participants on the improvement of the technique and the control of the emotions during the next examination of the images of tennis service. A chronometer (Chronomètre numérique iSport JG021 Pro) was equipped for each athlete to control the duration of the mental simulation of the ten tennis service tests of each session. Two coaches, who were considered experts in motor imagery training, were responsible for leading the interventions for the IMG and GC groups. These coaches assisted in the twelve IMG motor training sessions or videos for the CG, with the principle of avoiding any bias between the groups.

**Figure 1:**
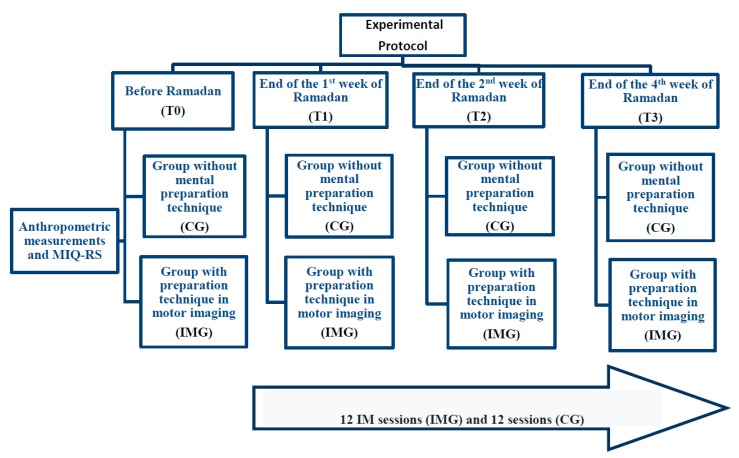
Experimental protocol adopted in the present study. Note. IMG = imagery training group; CG = control group; MIQ-RS = Motion Imagery Questionnaire.

The performance of tennis service (in terms of accuracy) was measured 48 hours before, during and at the end of the four weeks of the intervention during the Ramadan fasting, as shown in Figure 1.

The evaluation of the tennis service performance was possible thanks to the expertise of the coach in his discipline and the procedure described by a previous study [17]. To summarize, the players performed a series of ten tennis services. They were reminded that the goal of each service is to achieve a powerful and accurate service, while focusing on getting a winning shot, that is, looking for the ace on the “T”. A type radar gun (sr3800-fp Radar vitesse Pistolet) was located behind the player, to record the speed of the ball during the execution of the service.

After this introduction, the participants warmed up before hitting ten services diagonally, aiming for the “T” in order to familiarize themselves with the task.

**Figure 2:**
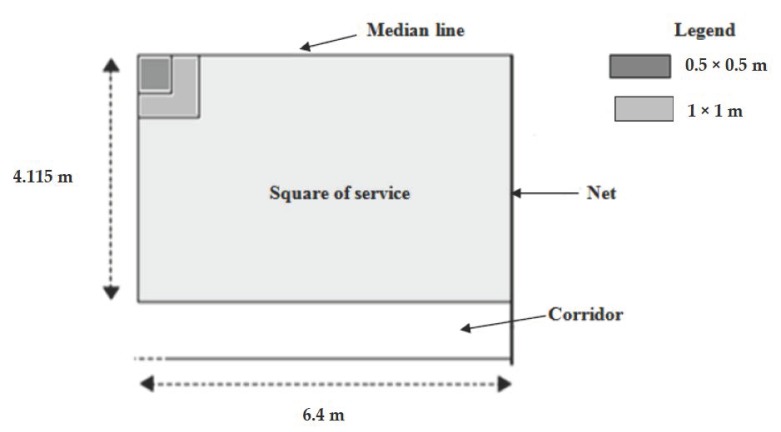
Service Performance Test (court scores).

A target with three zones has been adjusted in the field to evaluate the accuracy of the service, as shown in Figure 2. The accuracy was assessed from the location of the bounce of the ball in the opposing service square. A rebound in the dimension zone (0.5 × 0.5 m), defined from the center line and the service square line, yielded five points; a rebound in the dimension zone (1 × 1 m) yielded 3 points; a rebound of the ball in the rest of the service square yielded 1 point; a bounce out of the service square yielded 0 points (Figure 2). Thus, the accuracy was quantified from the score based on the sum of the points obtained at the end of the 10 services performed (the higher the score, the better the accuracy), while the speed of the ball measured during the execution of the service was computed as the average speed of all the shots. The performance of the tennis service was the product of accuracy and speed of the ball (accuracy × average speed of all balls (km/h)).

To evaluate the imaginative capacity of athletes, the Motion Imaging Questionnaire—Second Revised Version (MIQ-RS) was used.

The MIQ-RS [26] is a questionnaire consisting of 14 items and two scales (visual motion imagery and kinesthetic imaging). There are seven items that concern kinesthetic imaging and seven items related to visual motion imagery. The actions to be executed, similar in each scale, concern the upper limb, the lower limb, the whole body, and actions of daily life. Each scale contains seven possible response modalities (on a 7-point Likert scale). This study found an internal consistency of 0.79 for MIQ-RS.

Measurements of height and body mass were made using a board and electronic scale (Tanita, Tokyo, Japan), respectively. Moreover, we standardized calorie and hydric intake for the three days preceding each testing period.

To examine our hypotheses regarding the effects of motor imaging training on accuracy in tennis service, during the Ramadan fasting, factorial analyzes were performed. The mean and standard deviation were used to describe all variables (tennis service performance, height, body mass, training years, age and MIQ-RS). Before proceeding with such analyses, the normality of the distributions was tested with the Shapiro-Wilk method. When the Shapiro-Wilk test was not significant (*p* > 0.05), the normality assumption was not violated. A two-way ANOVA with repeated measures of both factors (Ramadan period (Before/During/After) × motor imagery (With or without)) was performed on the tennis service performance variables.

## 3. Results

The basic descriptive data for age, height, number of years of training and MIQ-RS are given in Table 1. It should be noted that no significant difference has been identified between IMG and CG before starting the intervention, which indicates the homogeneity of the groups. Table 3 shows the anthropometric values of the two groups (IMG and CG) before, at the beginning, in the middle and at the end of the Ramadan fasting. It should be noted that no significant difference was identified for body mass and body mass index (BMI) at T1 compared to T0. However, significant differences were observed in T2 and T3 compared to T0.

Two-way ANOVA with repeated measurements performed on body mass revealed a significant interaction of (Ramadan Period (Before/at the beginning/in the middle/at the end) × body mass), (F_(3.108)_ = 5.99; *p* = 0.017; $\eta_{p}^{2}$ = 0.08). The decrease in BMI was recorded only at the end of Ramadan. Statistical analysis showed a significant main effect (F_(3.108)_ = 5.44; *p* = 0.023; $\eta_{p}^{2}$ = 0.081).

Two-way ANOVA with repeated measurements, performed on the accuracy of the service revealed a significant interaction of (Ramadan Period (Before/at the beginning/in the middle/at the end) × motor imagery (With or without)), (F_(3.108)_ = 35.43; *p* < 0.001; $\eta_{p}^{2}$ = 0.496).

**Figure 3:**
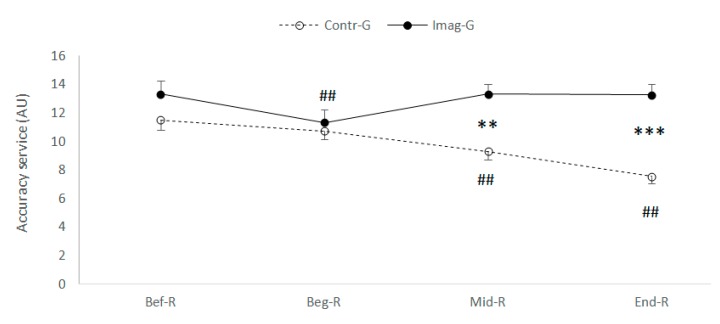
Mean and standard deviation of service accuracy, Before, During and at the end of Ramadan for both groups (CG vs. IMG). **, *** Significantly different from Contr-G at *p* < 0.01 and *p* < 0.001 respectively. ^##^ Significantly different from Bef-R at *p* < 0.001 respectively.

Fisher’s LSD post-hoc tests revealed that: (a) in the motor imaging condition, the accuracy of the service was lower at T1 compared to T0 (*p* < 0.001) (Figure 3), the service accuracy measured at T2 and at T3 did not differ from that measured before Ramadan (T0), (b) in training condition without motor imaging during Ramadan, the analyses revealed a decrease in the performance of service accuracy at T2 and T3 compared to T0 (*p* < 0.001). With respect to the difference between the groups, the accuracy of service was higher for the IMG compared to the GC at T2 and T3 (F_(1.36)_ = 10.52; *p* = 0.003; $\eta_{p}^{2}$ = 0.226), as shown in Figure 3.

**Figure 4:**
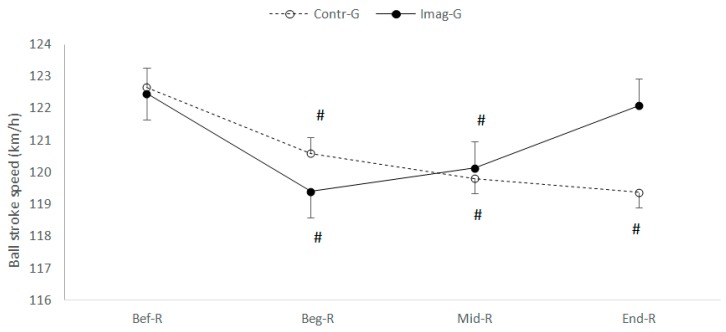
Mean and standard deviation of the ball stroke speed, before, during and at the end of Ramadan for both groups (CG vs. IMG). ^#^ Significantly different from Bef-R at *p* < 0.001.

Two-way ANOVA with repeated measurements performed on the ball stroke velocity revealed a significant interaction of (Ramadan Period (Before/at the beginning/in the middle/at the end) × motor imagery (With or without)) (F_(3.108)_ = 62.38; *p* < 0.001; $\eta_{p}^{2}$ = 0.634). Fisher’s LSD post-hoc tests revealed that: (a) in the motor imaging condition, the bullet velocity was lower at T1 and at T2 than at T0 (*p* < 0.001) (Figure 4), whereas, at T3 did not differ from that measured before Ramadan (T0), (b) under the condition of training without motor imaging during Ramadan, the analyses revealed a decrease in the performance of the ball travel speed at T1, T2 and T3 with respect to T0 (*p* < 0.001).

Regarding the difference between the groups, no significant difference was identified for the ball stroke speed (F_(1.36)_ = 0.19; *p* = 0.667; $\eta_{p}^{2}$ = 0.005), as shown in Figure 4.

**Figure 5:**
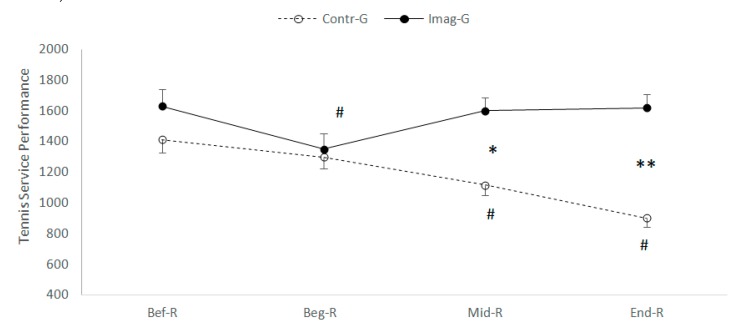
Mean and standard deviation of service performance, before, during and at the end of Ramadan for CG vs. IMG. *, ** Significantly different from Contr-G at *p* < 0.01, *p* < 0.001 respectively; ^#^ significantly different from Bef-R at *p* < 0.001.

Two-way ANOVA with repeated measurements, performed on the service performance (accuracy × ball run speed), revealed a significant interaction of the (Ramadan Period (Before/at the beginning/in the middle/at the end] × motor imagery (With or without) (F_(3.108)_ = 38.47; *p* < 0.001; $\eta_{p}^{2}$ = 0.517). Fisher’s LSD post-hoc tests revealed that: (a) in the motor imaging condition, the service performance was lower at T1 compared to T0 (*p* < 0.001) (Figure 5), whereas, at T2 and at T3 did not differ from that measured before Ramadan at T0, (b) under training condition without motor imaging during Ramadan. The analyses revealed a decrease in T2 service performance and T3 with respect to T0 (*p* < 0.001).

With regard to the difference between the groups, the service performance was higher for IMG compared to GC at T2 and T3 (F_(1.36)_ = 10.79; *p* = 0.002; $\eta_{p}^{2}$ = 0.231) (Figure 5).

## 4. Discussion

The aim of this study was to analyse the effects of motor imagery training on the performance of tennis service during the month of Ramadan among fasting male tennis players. Two hypotheses have been formulated: the first concerned the negative effects of Ramadan fasting on the performance of tennis service (in terms of accuracy and speed) and the second the feasibility of utilizing motor imagery training programs to counteract/mitigate the detrimental effects of the Ramadan fasting on the performance of tennis service (in terms of accuracy and speed).

Regarding the first hypothesis, our results revealed a decrease in the performance of tennis service (in terms of accuracy and speed) for CG at T2 and T3 compared to T0, confirming other studies showing a decline in physical performance during the month of Ramadan. Indeed, several authors have reported a decrease in performance in short-term trials [4,7,29,30], endurance [7,30,31,32] or repeated sprints [33,34]. A decrease in voluntary maximal contraction of the right elbow flexors for pilots was also reported [35], as well as a decrease in the maximum power of the arms and legs as measured by means of a load-speed test among a sample of physical education students [4]. This decrease in performance may be partially attributed to changes/disruptions in the rhythms of life during the month of Ramadan, such as the habits of consuming a large meal after sunset and a meal before dawn [36]. This amount of food consumed in the evening is likely to prevent falling asleep [37]. In addition, during this month, Muslims tend to go to bed later [38,39], which reduces sleep time [40]. The importance of sleep for athletic performance is well documented in the scholarly literature [41,42]. The delay in sleep and the reduction in sleep duration have been associated with a nocturnal increase in central temperature, possibly related to late meal times, which could lead to partial sleep deprivation [43].

There is an increase in daytime sleepiness during the month of Ramadan, associated with changes in the circadian rhythm of central temperature and metabolic changes associated with fasting [44]. Indeed, sleep is a key factor in optimizing training, competitions and recovery [42]. However, sleep is poorly described among athletes, especially during the month of Ramadan where significant changes in circadian rhythms are likely to occur [45]. Studies examining the sleep patterns of sportsmen fasting during the month of Ramadan remain scarce, especially for tennis players. 

The decrease in athletic performance could also be partially attributed to hypo-hydration during training due to the restriction of water intake during Ramadan [32,46,47]. Hypo-hydration may also be responsible for the decrease in performance of tennis service observed during the month of Ramadan. In this context, a study [33] showed a decrease in body weight in the afternoon compared to the morning during the Ramadan fasting. This reduction could be attributed to a loss of body water. Similarly, another study [32] observed severe dehydration associated with the Ramadan fasting, with a significant reduction in body mass. Further, dehydration has been shown to be detrimental to physical performance [37]. The negative effects of hypo-hydration on athletic performance are well documented in the literature [2]. Heat and humidity are other factors that can affect sports performance [48]. Indeed, athletes who fast during their training or competitions lasting more than 30 minutes may face particular difficulties when the ambient temperature is high [13]. In the present investigation, we recorded an average temperature of 29.13 °C and a 61.46% humidity in the afternoon during training in the month of Ramadan in 2017. With regard to the second hypothesis, results showed that, during the Ramadan fasting, motor imaging training programs can counteract/mitigate the negative effects of fasting on the performance of tennis service (in terms of accuracy and speed) confirming our hypothesis. Further, this finding is consistent with previous studies showing an improvement in the performance of physical tasks through a motor imaging training program [17,18,20]. The accuracy of tennis service as measured in our investigation was lower at the beginning of Ramadan (T1) and in the middle of Ramadan (T2) than at the beginning and at the end of Ramadan, respectively at T0 and T3, while the service accuracy measured at the end of Ramadan (T3) did not differ from that measured before Ramadan (T0). This result is corroborated by the difference between the CG and the IMG, in terms of the accuracy of service. The value was higher for IMG compared to CG at T2 and T3, which suggests that the motor imaging training program did not improve the performance of tennis service (as evaluated by its accuracy) at the beginning of the month of Ramadan (T1). The improvement began to be noted after six training sessions in motor imaging, i.e., at T2. This is in agreement with a study [17], which has demonstrated that the effects of motor imagery training on the performance in terms of speed and accuracy of the ball among young tennis players did not attenuate after a single high-intensity workout. On the other hand, another study [21] showed an improvement in service performance (in terms of accuracy and speed) among French tennis players after six weeks of training in motor imaging.

Concerning the timing of integration of the motor imaging program in the usual training sessions, we incorporated the motor imaging module within the training program just before the physical and technical training. This is in agreement with a number of studies [49,50], which demonstrated that physical fatigue affects the creation of mental images, which could have a negative impact on the effectiveness of the motor imaging training program for swimmers. In contrast, other studies have shown beneficial effects of motor imaging training either after or during physical training sessions [12,17,27,28]. 

Our study showed that motor imagery training can represent an effective interventional strategy in the month of Ramadan for tennis players, which can counteract/mitigate the negative effects of fasting on the physical and mental performance. This could be explained by an increase in neuronal activity at the level of several areas of the brain responsible for the execution of motor control. Motor imagery training would lead to an improved performance through the integration of motor imagery and mental rehearsal in physical and technical training throughout the month of Ramadan. In this sense, a study [51] has stated that some neurophysiologic mechanisms in the brain may explain the improvement in motor performance, while at the muscle activation level, mental imagery training can have a positive effect on performance. A study [22] has shown that the impact of a motor imaging training program on target muscle activation levels may have an effect on changes in neuronal muscle control, as it can have a beneficial effect on the muscular coordination and on the musculature in general, through the (partial or total) inhibition of the activity of the antagonistic muscles during the exercise. This can explain the improvement in the accuracy of the IMG tennis service at T2 and T3 with respect to T1 time-point. Strength and muscular power are necessary to have a fast and powerful service in terms of speed and accuracy of execution. 

Other studies [52,53] have shown that neuronal adaptations can increase muscle strength gains. Therefore, it has been suggested that a motor imaging-based training program can increase the physical performance based on the distal and proximal muscles of the lower and upper limbs. Similarly, it has been shown that a training program incorporating motor imagery modules can largely explain the gains in muscle strength [54], which is at the origin of adaptations of the neuronal components, thus explaining the improvement in the speed of stroke of the ball in tennis service for the IMG at the end of Ramadan (T3) compared to the beginning and the middle of the Ramadan fasting, respectively T1 and T2. 

## 5. Limitations of the Study

Despite the interesting results reported in this study, our present investigation suffers from a number of limitations that should be acknowledged. For instance, we have not evaluated the tennis service technique that was employed by the groups (IMG and CG), which can influence performance. The training sessions and the evaluation of the performance of tennis service were conducted during the afternoon of the month of Ramadan, because of the change in the training schedule during this month, while the majority of tennis matches took place in the morning, which is another aspect that could likely influence performance. Furthermore, we did not collect precise data concerning the hours of sleep and, despite the fact that we measured body weight, this remains an indirect proxy of the level of hydration. Finally, the lack of data of the electrical activity of the brain may represent another limitation of the present investigation.

Thus, the results of the current study should be treated with caution, and subsequent studies should be undertaken and take into account the tennis service technique utilized, the timing of the training sessions and the evaluation of the performance of tennis service. All these variables should be assessed at the same time as the matches and, finally, the muscular activity during training sessions in motor imaging should be analyzed by means of electromyography.

## 6. Conclusions

This study represents a first attempt to examine the effects of motor imagery training on the performance of tennis service during the month of Ramadan among fasting male tennis players. Results showed that motor imagery training could be an effective strategy for optimizing the performance of tennis service during the Ramadan fasting and could counteract/mitigate the negative and detrimental effects of the fasting on the performance of tennis service.

Thus, from a practical point of view, the incorporation of a motor imaging training program during the physical and technical training sessions in the month of Ramadan seems to be an adequate interventional strategy. However, due the above-mentioned shortcomings, further research in the field is warranted.

## Figures and Tables

**Table 1:** Mean and standard deviation of descriptive research variables.

Variables	IMG	CG	*p*
	Mean (SD)	Mean (SD)	
**Age (years)**	16.9 ± 0.6	16.7 ± 0.8	0.34
**Body mass (kg)**	67.4 ± 5.9	66.2 ± 9.3	0.32
**Height (m)**	1.8 ± 0.1	1.8 ± 0.1	0.36
**Number of years of training (years)**	5.4 ± 1.3	5.7 ± 1.2	0.28
**MIQ-RS**			
**VIM**	5.4 ± 0.6	5.4 ± 0.8	0.36
**KIM**	5.3 ± 0.7	5.3 ± 0.6	0.38

Note. SD = standard deviation; MIQ-RS = Movement Imagery Questionnaire; VIM = visual motor imagery; KMI = kinesthetic motor imagery; IMG = imagery training group; CG = control group.

**Table 2:** Averages (±SD) of daily calorie intake and percentages of carbohydrates, fats and proteins recorded before and during the month of Ramadan.

Variables	Bef-R	Ramadan
	Mean (SD)	Mean (SD)
**Calorie intake (kcal/day)**	3147 ± 141.3	3133 ± 182.2
**Carbohydrate (%)**	50.4 ± 5.6	50.1 ± 3.8
**Lipids (%)**	32.1 ± 5.8	30.6 ± 3.8
**Protein (%)**	19.9 ± 2.6	20.2 ± 3.8

Note. SD = standard deviation; Bef-R = before Ramadan.

**Table 3:** Averages (±SD) of body mass and BMI recorded before and during Ramadan.

Parameters	Groups	Bef-R	Beginning-R	Mid-R	End-R	Ramadan × Group Interaction		
						F_(3.108)_	*p*-Value	$\eta_{p}^{2}$
		Mean (SD)	Mean (SD)	Mean (SD)	Mean (SD)			
**Body mass (kg)**	**CG**	66.2 ± 9.3	65.9 ± 8.7	64.2 ± 7.9 ^#^	63.8 ± 9.1 ^#^	5.99	0.017	0.08
	**IMG**	67.4 ± 5.9	67.2 ± 5.2	65.3 ± 4.9 ^#^	65.1 ± 5.3 ^#^			
**BMI (kg/M^2^)**	**CG**	21.4 ± 4.7	21.3 ± 4.3	20.7 ± 3.9 ^#^	20.6 ± 4.6 ^#^	5.44	0.023	0.08
	**IMG**	21.5 ± 3.6	21.4 ± 3.1	20.8 ± 3.3 ^#^	20.8 ± 3.0 ^#^			

Note. SD = standard deviation; IMG = imagery training group; CG = control group; BMI = body mass index; ^#^ Significantly different from Bef-R at *p* < 0.05.

## References

[1] Maughan R.J. (2010). Fasting and sport: An introduction. Br. J. Sports Med..

[2] Judelson D.A., Maresh C.M., Anderson J.M., Armstrong L.E., Casa D.J., Kraemer W.J. (2007). Hydration and muscular performance: Does fluid balance affects strength, power and high-intensity endurance?. Sports Med..

[3] Baklouti H., Chtourou H., Aloui A., Chaouachi A., Souissi N. (2015). Effect of active warm-up duration on morning short-term maximal performance during Ramadan. Libyan J. Med..

[4] Bouhlel H., Shephard R.J., Gmada N., Aouichaoui C., Peres G., Tabka Z., Bouhlel E. (2013). Effect of Ramadan observance on maximal muscular performance of trained men. Clin. J. Sport Med..

[5] Chtourou H., Hammouda O., Souissi H., Chamari K., Chaouachi A., Souissi N. (2011). The effect of Ramadan fasting on physical performances, mood state and perceived exertion in young footballers. Asian J. Sports Med..

[6] Chennaoui M., Desgorces F., Drogou C., Boudjemaa B., Tomaszewski A., Depiesse F., Burnat P., Chalabi H., Gomez Merino D. (2009). Effects of Ramadan fasting on physical performance and metabolic, hormonal, and inflammatory parameters in middle-distance runners. Appl. Physiol. Nutr. Metab..

[7] Zerguini Y., Kirkendall D., Junge A., Dvorak J. (2007). Impact of Ramadan on physical performance in professional soccer players. Br. J. Sports Med..

[8] Carvalho J., Araújo D., González L.G., Iglesias D. (2011). El entrenamiento de la toma de decisiones en el tenis: ¿qué fundamentos científicos se pueden aplicar en los programas de entrenamiento. Rev. Psicol. Deporte.

[9] López-Samanes Á.G., Pallarés J., Pérez-López A., Mora-Rodríguez R., Ortega J.F. (2018). Hormonal and neuromuscular responses during a singles match in male professional tennis players. PLoS ONE.

[10] Fernandez-Fernandez J., Mendez-Villanueva A., Pluim B. (2006). Intensity of tennis match play. Br. J. Sports Med..

[11] Brody H. (2003). Serving strategy. ITF Coach. Sport Sci. Rev..

[12] Pereira T.J.C., Nakamura F.Y., Jesus M.T., Vieira L.R.L., Misuta M.S., Barros R.M.L., Moura F.A. (2016). Analysis of the distances covered and technical actions performed by professional tennis players during official matches. J. Sports Sci..

[13] Maughan R.J., Fallah J., Coyle E.F. (2010). The effects of fasting on metabolism and performance. Br. J. Sports Med..

[14] Hayes M.J., Spits D.R., Watts D.G., Kelly V.G. (2018). The relationship between tennis serve velocity and select performance measures. J. Strength Cond. Res..

[15] Whiteside D., Reid M. (2017). Spatial characteristics of professional tennis serves with implications for serving aces: A machine, learning approach. J. Sports Sci..

[16] Kanthack T.F.D., Bigliassi M., Vieira L.F., Altimari L.R. (2014). Acute effect of motor imagery on basketball players’ freethrow performance and self-efficacy. Rev. Bras. Cineantropom. Desempenho Hum..

[17] Guillot A., DiRienzo F., Pialoux V., Simon G., Skinner S., Rogowski I. (2015). Implementation of motor imagery during specific aerobic training session in young tennis players. PLoS ONE.

[18] Fortes L.D., Almeida S.S., Nascimento-Júnior J.R., Fiorese L., Lima-Junior D.D., Ferreira M.E. (2019). Effect of motor imagery training on tennis service performance in young tennis athletes. J. Sport Psychol..

[19] DiRienzo F., Blache Y., Kanthack T.F.D., Monteil K., Collet C., Guillot A. (2015). Short-term effects on integrated motor imagery practice on muscle activation and force performance. Neuroscience.

[20] Battaglia C., D’Artibale E., Fiorilli G., Piazza M., Tsopani D., Giombini A., Calcagno G., di Cagno A. (2014). Use of video observation and motor imagery on jumping performance in national rhythmic gymnastics athletes. Hum. Mov. Sci..

[21] Guillot A., Desliens S., Rouyer C., Rogowski I. (2013). Motor imagery and tennis serve performance: The external focus efficacy. J. Sports Sci. Med..

[22] Slimani M., Tod D., Chaabene H., Miarka B., Chamari K. (2016). Effects of mental imagery on muscular strength in healthy and patient participants: A systematic review. J. Sports Sci. Med..

[23] Ridderinkhof K.R., Brass M. (2015). How kinesthetic motor imagery works: A predictive-processing theory of visualization in sports and motor expertise. J. Physiol. Paris.

[24] Sureshet KP., Chandrashekara S. (2012). Sample size estimation and power analysis for clinical research studies. J. Hum. Reprod. Sci..

[25] Wang Z., Wang S., Shi F.Y., Guan Y., Wu Y., Zhang Z., Shen C., Zeng W., Wang D.H., Zhang J. (2014). The effect of motor imagery with specific implement in expert badminton player. Neuroscience.

[26] Loison B., Moussaddaq A.S., Cormier J., Richard I., Ferrapie A.L., Ramond A., Dinomais M. (2013). Translation and validation of the french Mouvement Imagery Questionnaire–Revised second version(MIQ-RS). Ann. Phys. Rehabil. Med..

[27] Fortes L.S., Lira H.A.A.S., Lima R.C.P., Almeida S.S., Ferreira M.E.C. (2016). Mental training generates positive effect on competitive anxiety of young swimmers?. Rev. Bras. Cineantropom. Desempenho Hum..

[28] Fortes L.S., Freitas-Júnior C.G., Paes P.P., Vieira L.F., Nascimento-Júnior J.R.A., Lima-Júnior D.R.A.A., Ferreira M.E.C. (2020). Effect of an eight-week imagery training programme on passing decision-making of young volley ball players. Int. J. Sport Exerc. Psychol..

[29] Chtourou H., Hammouda O., Chaouachi A., Chamari K., Souissi N. (2012). The effect of time-of-day and Ramadan fasting on anaerobic performances. Int. J. Sports Med..

[30] Meckel Y., Ismaeel A., Eliakim A. (2008). The effect of the Ramadan fast on physical performance and dietary habits in adolescent soccer players. Eur. J. Appl. Physiol..

[31] Chaouachi A., Chamari K., Roky R., Wong D.P., Mbazaa A., Bartagi Z., Amri M. (2008). Lipid profiles of judo athletes during Ramadan. Int. J. Sports Med..

[32] Sweileh N., Schnitzler A., Hunter G.R., Davis B. (1992). Body composition and energy metabolism in resting and exercising Muslims during Ramadan fast. J. Sports Med. Phys. Fit..

[33] Aloui A., Chaouachi A., Chtourou H., Wong D.P., Haddad M., Chamari K., Souissi N. (2013). Effects of Ramadan on the diurnal variations of repeated-sprint performance. Int. J. Sports Physiol. Perform..

[34] Girard O., Farooq A. (2012). Effects of Ramadan fasting on repeated sprint ability in young children. Sci. Sports.

[35] Bigard A.X., Boussif M., Chalabi H., Guezennec C.Y. (1998). Alteration sin muscular performance and orthostatic tolerance during Ramadan. Aviat. Space Environ. Med..

[36] Ibrahim W.H., Habib H.M., Jarrar A.H., AlBaz S.A. (2008). Effect of Ramadan fasting on markers of oxidative stress and serum biochemical markers of cellular damage in healthy subjects. Ann. Nutr. Metab..

[37] Waterhouse J., Hudson P., Edwards B. (2010). Effects of music tempo upon sub maximal cycling performance. Scand. J. Med. Sci. Sports.

[38] Afifi Z.E. (1997). Daily practices, study performance and health during the Ramadan fast. J. R. Soc. Health.

[39] BaHammam A. (2005). Assessment of sleep patterns, day time sleepiness, and chronotype during Ramadan in fasting and non fasting individuals. Saudi Med. J..

[40] Bogdan A., Bouchareb B., Touitou Y. (2001). Ramadan fasting alters endocrine and neuro endocrine circadian patterns. Meal-time as asynchronizer in humans?. Life Sci..

[41] Mah C.D., Mah K.E., Kezirain E.J., Dement W.C. (2011). The effects of sleep extension on the athletic performance of collegiate basket ball players. Sleep.

[42] Samuels C. (2008). Sleep, recovery, and performance: The new frontier in high-performance athletics. Neurol. Clin..

[43] Roky R., Chapotot F., Hakkou F., Benchekroun M.T., Buguet A. (2001). Sleep during Ramadan intermittent fasting. J. Sleep Res..

[44] Roky R., Chapotot F., Ben chekroun M.T., Benaji B., Hakkou F., Elkhalifi H., Buguet A. (2003). Day time sleepiness during Ramadan intermittent fasting: polysomnographic and quantitative waking EEG study. J. Sleep Res..

[45] Reilly T., Waterhouse J. (2007). Altered sleep-wake cycles and food intake: The Ramadan model. Physiol. Behav..

[46] Mustafa K.Y., Mahmoud N.A., Gumaa K.A., Gader A.M. (1978). The effects of fasting in Ramadan. 2. Fluid and electrolyte balance. Br. J. Nutr..

[47] Ramadan J. (2002). Does fasting during Ramadan alter body composition, blood constituents and physical performance?. Med. Princ. Pract..

[48] Matta M.O., Figueiredo J.F.B., Garcia E.S., Seabra A.F.T. (2014). Morphological, maturational, functional and technical profile of young Brazilian soccer players. Rev. Bras. Cineantropom. Desempenho Hum..

[49] Demougeot L., Papaxanthis C. (2011). Muscle fatigue affects mental simulation of action. J. Neurosci..

[50] Di Rienzo F., Collet C., Hoyek N., Guillot A. (2012). Selective effect of physical fatigue on motor imagery accuracy. PLoS ONE.

[51] Ruffino C., Papaxanthis C., Lebon F. (2016). Neural plasticity during motor learning with motor imagery practice: Review and perspectives. Neuroscience.

[52] Schoenfeld B.J., Ogborn D., Krieger J.W. (2016). Dose-response relationship between weekly resistance training volume and increases in muscle mass: A systematic review and meta-analysis. J. Sports Sci..

[53] Fontani G., Migliorini S., Benocci R., Facchini A., Casini M., Corradeschi F. (2007). Effect of mental imagery on the development of skilled motor actions. Percept. Mot. Skills.

[54] Yao W.X., Ranganathan V.K., Allexandre D., Siemionow V., Yue G.H. (2013). Kinesthetic imagery training of forceful muscle contractions increases brain signal and muscle strength. Front. Hum. Neurosci..

